# ordinalbayes: Fitting Ordinal Bayesian Regression Models to High-Dimensional Data Using R

**DOI:** 10.3390/stats5020021

**Published:** 2022-04-15

**Authors:** Kellie J. Archer, Anna Eames Seffernick, Shuai Sun, Yiran Zhang

**Affiliations:** 1 Division of Biostatistics, College of Public Health, The Ohio State University, Columbus, OH 43210, USA; 2 Amgen Inc., 1 Amgen Center Dr, Thousand Oaks, CA 91320, USA

**Keywords:** cumulative logit, penalized models, LASSO, variable inclusion indicators, spike-and-slab

## Abstract

The stage of cancer is a discrete ordinal response that indicates the aggressiveness of disease and is often used by physicians to determine the type and intensity of treatment to be administered. For example, the FIGO stage in cervical cancer is based on the size and depth of the tumor as well as the level of spread. It may be of clinical relevance to identify molecular features from high-throughput genomic assays that are associated with the stage of cervical cancer to elucidate pathways related to tumor aggressiveness, identify improved molecular features that may be useful for staging, and identify therapeutic targets. High-throughput RNA-Seq data and corresponding clinical data (including stage) for cervical cancer patients have been made available through The Cancer Genome Atlas Project (TCGA). We recently described penalized Bayesian ordinal response models that can be used for variable selection for over-parameterized datasets, such as the TCGA-CESC dataset. Herein, we describe our ordinalbayes R package, available from the Comprehensive R Archive Network (CRAN), which enhances the runjags R package by enabling users to easily fit cumulative logit models when the outcome is ordinal and the number of predictors exceeds the sample size, *P* > *N*, such as for TCGA and other high-throughput genomic data. We demonstrate the use of this package by applying it to the TCGA cervical cancer dataset. Our ordinalbayes package can be used to fit models to high-dimensional datasets, and it effectively performs variable selection.

## 1. Introduction

Despite the advent of HPV vaccinations and effective screening programs, globally, cervical cancer is the fourth most common cancer among women [1]. The estimated number of new cases in 2020 is 604,127 with 341,831 deaths [2]. The stage of cervical cancer, as outlined in the International Federation of Gynecology and Obstetrics (FIGO) guidelines, is based on physical examinations, endoscopic procedures, and imaging. Specifically, the FIGO stage is based on the size and depth of the tumor as well as the level of spread [3]. It is important that the stage, a discrete ordinal response, be correct as it is used to guide treatment planning, counsel patients with respect to prognosis, and to determine whether the patient meets eligibility criteria for available clinical trials or other research studies [4,5]. Unfortunately, there is still debate as to whether surgical or non-invasive radiological modalities for identifying parametrial and lymph node involvement is preferred when staging a patient [4]. Thus, it is clinically relevant to identify molecular features from high-throughput genomic assays that are associated with the stage of cervical cancer to elucidate pathways related to tumor aggressiveness, identify improved molecular features that may be useful for staging, and identify therapeutic targets.

Penalized frequentist models have been widely applied when analyzing high-dimensional data. Such models were initially described for linear [6] and logistic [7] regression and subsequently for ordinal response models [8–10]. However, when applying penalized frequentist models, the penalty parameter, or vector of parameters in the case of elastic net, must be selected by the analyst. As a result, the coefficient estimates from the resulting model are conditional on that penalty parameter. For that reason, penalized Bayesian models were developed for the linear [11–14] and logistic [15–17] regression settings. We also recently described penalized Bayesian models for the ordinal response setting [18] and demonstrated that our penalized Bayesian cumulative logit model has improved variable selection performance when compared to penalized frequentist cumulative logit models [19].

Herein, we describe our ordinalbayes R package, which enhances the runjags R package [20] by enabling users to easily fit penalized Bayesian cumulative logit models. The ordinalbayes function can be used to fit LASSO, normal spike-and-slab, double exponential spike-and-slab, and regression-based variable inclusion indicator Bayesian models. Variable selection can be performed using the Bayes factor or using the posterior distributions of the variable inclusion indicators directly. In the following sections, we describe our implementation and describe the syntax required for each of our Bayesian models. We then illustrate the functions in the ordinalbayes R package using two examples where we were interested in identifying transcripts important to predicting the FIGO stage in cervical cancer patients using high-throughput gene expression data. A small example is provided in Appendix A.

## 2. Materials and Methods

### 2.1. Ordinal Bayesian Models and R Syntax

We previously described four penalized cumulative logit Bayesian models that can be fit when the covariate space is high-dimensional [18]. This includes a regression-based variable inclusion indicator ordinal model, a LASSO ordinal model, a normal spike-and-slab ordinal model, and a double exponential spike-and-slab ordinal model. To introduce our penalized cumulative logit Bayesian models, we let *Y*_1_, … ,*Y*_*n*_ represent the ordinal responses for *n* subjects, which can take on one of 1, … , *K* ordinal response levels, with *K* representing the number of ordinal levels. Let ***x***_*i*_ = (*x*_*i*1_, *x*_*i*2_, … , *x*_*iP*_)′ represent the vector of covariates for subject *i*, where *P* represents the number of predictors. When assuming proportional odds, the effect of each covariate is constant across all ordinal response levels such that the slope for the ordinal responses are parallel. For each ordinal level *k* = 1, 2, … , *K* − 1, let ***β*** = (*β*_1_, *β*_2_ … , *β*_*P*_)′ denote a vector of unknown regression coefficients. The cumulative logit model is

$$\log\left[\frac{Pr\left(Y_{i}\leq k|x_{i}\right)}{Pr\left(Y_{i}>k|x_{i}\right)}\right]=\alpha_{k}-\beta^{\prime }x_{i},\;k=1,2,\ldots ,K-1,$$

where *Pr*(*Y*_*i*_ ≤ *k*|***x***_*i*_) is the cumulative probability of the event *Y*_*i*_ ≤ *k* given ***x***_*i*_. The thresholds differentiate between the *K* ordinal levels and must satisfy the constraint −∞ = *α*_0_ < *α*_1_ <*α*_2_ < ··· < *α*_*K*−1_ <*α*_*K*_ = ∞.

Herein, we describe our ordinalbayes package that enhances the functionality of the runjags package by providing functions specific to fitting these four penalized ordinal Bayesian models and extracting results of interest. We also provide an overview of each model. Tables summarizing the package functions and syntax appears in Appendix C.

The primary function for model fitting in the ordinalbayes package is ordinalbayes. The function arguments are


function (formula, data, x = NULL, subset, center = TRUE, scale = TRUE,
a = 0.1, b = 0.1, model = “regressvi”, gamma.ind = “fixed”,
pi.fixed = 0.05, c.gamma = NULL, d.gamma = NULL, alpha.var = 10,
sigma2.0 = NULL, sigma2.1 = NULL, coerce.var=10, lambda0 = NULL,
adaptSteps = 5000, burnInSteps = 5000, nChains = 3, numSavedSteps = 9999,
thinSteps = 3, parallel = TRUE, seed = NULL, quiet = FALSE)


This function accepts a model formula that specifies the ordinal response on the left-hand side of the equation and any unpenalized predictor variable(s) on the right-hand side of the equation. Unpenalized predictors are variables such as age that we include in the model without applying any shrinkage of their corresponding parameter estimates. When unpenalized predictors are included as covariates in the model, the user can specify the variance associated with the corresponding model parameters (default coerce.var = 10). If no unpenalized predictor variables are included, the model formula should be y ~ 1 (representing the intercept). The user can subset the data.frame prior to model fitting, for example, subset=(race ==“white”). To specify the penalized covariates in the model, the user should pass the data.frame to the x parameter, indicating the relevant columns of covariates. By default, the penalized covariates are centered (center = TRUE) and scaled (scale = TRUE).

The selected parameters are initialized prior to updating through MCMC. For one chain, the *k* − 1 ordinal thresholds, *α*_*k*_, are initialized to the logit of the cumulative response probabilities, which is equivalent to the estimated *k* − 1 thresholds in an intercept-only model

$$\alpha_{k}=\log\left(\frac{\sum_{i=1}^{n}\sum_{m=1}^{k}Y_{ik}/n}{1-\sum_{i=1}^{n}\sum_{m=1}^{k}Y_{ik}/n}\right).$$

For multiple chains, initial values for the *α*_*k*_ terms for chains beyond the first chain are sampled from a Normal(0, 0.5) distribution and then sorted to impose the *α*_1_ < ··· <*α*_*k*−1_ order restriction. Within the MCMC, the *α*_*k*_ terms are sampled from a Normal $\left(0,\sigma_{\alpha_{k}}^{2}\right)$, and users can adjust the variance by specifying alpha.var (default 10 such that the precision is 0.10). All penalized coefficients (*β*_*j*_ for *j* = 1, … , *P*) are initialized to zero.

Other relevant parameters common to all model types include: nChains, the number of parallel chains for the model (default 3); adaptSteps, the number of iterations for adaptation (default 5000); burnInSteps, the number of iterations of the Markov chain to run (default 5000); numSavedSteps, the number of saved steps per chain (default 9999); and thinSteps, the thinning interval for monitors (default 3). Provided the user will be running the model on a machine with multiple processors, the computational speed can be improved by running the chains in parallel by specifying parallel = TRUE. When parallel = TRUE, runjags executes the MCMC sampling using nChains parallel processors. To ensure the user can obtain reproducible results, seed accepts an integer that is used to set the random seed. The output from JAGS can be suppressed by specifying quiet = TRUE. The user can fit one of four available Bayesian models. A list of the parameters the user can set for all four models is provided in Table A1. Following Section 2.1.1, which describes applying ordinalbayes to Bioconductor objects, each of the four models is described along with the relevant arguments that must be specified by the user. A list of the parameters the user needs to set for each specific model is provided in Table A2.

#### 2.1.1. Use with Bioconductor Objects: SummarizedExperiment and ExpressionSet

When analyzing data processed using the DESeq2 Bioconductor package, the genomic feature object is of class DESeqTransform, which is a SummarizedExperiment, and therefore, the phenotypic data are accessed using the colData extractor function. When analyzing data processed using packages that structure the genomic feature object as a Biobase ExpressionSet, the phenotypic data are accessed using the pData extractor function. Therefore, in the ordinalbayes call, data should be either a colData() or pData() call to the genomic feature object. Again, the ordinalbayes function accepts a model formula that specifies the ordinal response on the left-hand side of the equation and any unpenalized predictor variable(s) from the phenotypic dataset on the right-hand side of the equation. If no unpenalized predictor variables are included, the model formula should be y ~ 1 (representing the intercept).

When specifying the penalized covariates in the model, the user should pass to the x parameter the appropriate call for extracting the genomic feature data from the object. For SummarizedExperiment objects, the genomic features to be penalized are accessed using the assay() extractor function. For ExpressionSet objects, the genomic features to be penalized are accessed using the exprs() extractor function. The user can also pass a matrix to x; however, the user needs to carefully verify that the observations in the x matrix are appropriately aligned to the phenotypic data. Note that the number of rows in both data and x should be the same, such that the transpose of assay or exprs should be supplied to x.

### 2.2. Regression-Based Variable Inclusion Indicator Ordinal Model

By default, the model that is fit is the regression-based variable inclusion indicator Bayesian model, specified by model = “regressvi”. This model takes the form

$$\log\left[\frac{Pr\left(Y_{i}\leq k|x_{i}\right)}{Pr\left(Y_{i}>k|x_{i}\right)}\right]=\alpha_{k}-\sum_{j=1}^{p}\gamma_{j}\beta_{j}x_{ij},\;\;\text{for}\;k=1,2,\ldots ,K-1\;\beta_{j}|\lambda \sim \mathrm{DE}(0,1/\lambda ),\;\;\text{for}\;j=1,\ldots ,p\;\lambda \sim \mathrm{Gamma}(a,b)\;\alpha_{k}\sim \mathrm{Normal}\left(0,\sigma_{\alpha_{k}}^{2}\right),\alpha_{1}<\alpha_{2}<\ldots <\alpha_{K-1},\;\;\text{for}\;\;k=1,2,\ldots ,K-1\;\gamma_{j}\sim \mathrm{Bernoulli}\left(\pi_{j}\right),\;\;\text{for}\;j=1,\ldots ,p\;\pi_{j}=t\;\text{or}\;\pi_{j}\sim \mathrm{Beta}(c,d),\;\;\text{for}\;j=1,\ldots ,p$$

and assumes the penalized coefficients are from a Laplace (or double exponential) distribution with parameter *λ* and that *λ* is from a Gamma distribution with parameters a and b. Based on our extensive simulations [19], model performance is not affected by choices of a and b, so we provide defaults of 0.1 for both. The variable inclusion indicator *γ*_*j*_ is assumed to follow a Bernoulli distribution with parameter *π*_*j*_. The user can use either a fixed constant prior (default) or a random prior. When using a fixed constant prior, the user must specify both gamma.ind=“fixed” and set pi.fixed to some constant in the (0, 1) interval (default is 0.05). Alternatively, a random prior for *π*_*j*_ is acheived by specifying both gamma.ind=“random” and parameter values (c.gamma and d.gamma) for the Beta distribution. Values of c.gamma and d.gamma should be selected such that the mean of the Beta distribution for the variable inclusion indicators corresponds to the anticipated proportion of covariates truly associated with the ordinal response, given by *c*/(*c* + *d*), while considering that the variance is given by

$$\frac{cd}{{\left(c+d\right)}^{2}(c+d+1)}.$$

If unpenalized coefficients are included in the model, their coefficients are ζ ~ Normal $\left(0,\sigma_{coerce\;}^{2}\right)$.

### 2.3. Lasso Ordinal Model

The LASSO Bayesian ordinal model can be fit by specifying model=“lasso”. This model assumes the penalized coefficients *β*_*j*_ for *j* = 1, … , *P* are from independent Laplace (or double exponential) distributions with parameter *λ* and that *λ* is from a Gamma distribution with parameters a and b.

$$\log\left[\frac{Pr\left(Y_{i}\leq k|x_{i}\right)}{Pr\left(Y_{i}>k|x_{i}\right)}\right]=\alpha_{k}-\sum_{j=1}^{p}\beta_{j}x_{ij},\;\;\text{for}\;k=1,2,\ldots ,K-1\;\beta_{j}|\lambda \sim \mathrm{DE}(0,1/\lambda ),\;\;\text{for}\;j=1,\ldots ,p\;\lambda \sim \mathrm{Gamma}(a,b)\;\alpha_{k}\sim \mathrm{Normal}\left(0,\sigma_{\alpha_{k}}^{2}\right),\alpha_{1}<\alpha_{2}<\ldots <\alpha_{K-1},\;\;\text{for}\;\;k=1,2,\ldots ,K-1$$

As previously mentioned, model performance is not affected by choices of a and b, so we provide defaults of 0.1 for both. If unpenalized coefficients are included in the model, their coefficients are $\zeta \sim \mathrm{Normal}\left(0,\sigma_{coerce\;}^{2}\right)$.

### 2.4. Normal Spike-and-Slab Ordinal Model

The normal spike-and-slab Bayesian ordinal model can be fit by specifying model=“normalss”. This model is given by

$$\log\left[\frac{Pr\left(Y_{i}\leq k|x_{i}\right)}{Pr\left(Y_{i}>k|x_{i}\right)}\right]=\alpha_{k}-\sum_{j=1}^{p}\beta_{j}x_{ij},\;\;\text{for}\;k=1,2,\ldots ,K-1\;\beta_{j}|\gamma_{j}\sim \left(1-\gamma_{j}\right)\times \mathrm{Normal}\left(0,\sigma_{0}^{2}\right)+\gamma_{j}\times \mathrm{Normal}\left(0,\sigma_{1}^{2}\right),\;\;\text{for}\;j=1,\ldots ,p\;\alpha_{k}\sim \mathrm{Normal}\left(0,\sigma_{\alpha_{k}}^{2}\right),\alpha_{1}<\alpha_{2}<\ldots <\alpha_{K-1},\;\;\text{for}\;\;k=1,2,\ldots ,K-1\;\gamma_{j}\sim \mathrm{Bernoulli}\left(\pi_{j}\right),\;\;\text{for}\;j=1,\ldots ,p\;\pi_{j}=t\;\text{or}\;\pi_{j}\sim \mathrm{Beta}(c,d),\;\;\text{for}\;j=1,\ldots ,p\text{.}\;$$

When fitting this model, the user is required to specify the variance for the spike $\left(\sigma_{0}^{2}\right)$ by setting sigma2.0 to a small positive value (e.g., 0.01) and variance for the slab $\left(\sigma_{1}^{2}\right)$ by setting sigma2.1 to a large positive value (e.g., 10). As with the regression-based variable inclusion indicator Bayesian model, the variable inclusion indicator *γ*_*j*_ is assumed to follow a Bernoulli distribution with parameter *π*_*j*_. The user can use either a fixed constant prior (default) or a random prior. When using a fixed constant prior, the user must specify both gamma.ind=“fixed” and set pi.fixed to some constant in the (0, 1) interval (default is 0.05). Alternatively, a random prior for *π*_*j*_ is acheived by specifying both gamma.ind=“random” and parameter values (c.gamma and d.gamma) for the Beta distribution. If unpenalized coefficients are included in the model, their coefficients are $\zeta \sim \mathrm{Normal}\left(0,\sigma_{coerce\;}^{2}\right)$.

### 2.5. Double Exponential Spike-and-Slab Ordinal Model

The double exponential spike-and-slab ordinal model can be fit by specifying model=“dess” and is given by

$$\log\left[\frac{Pr\left(Y_{i}\leq k|x_{i}\right)}{Pr\left(Y_{i}>k|x_{i}\right)}\right]=\alpha_{k}-\sum_{j=1}^{p}\beta_{j}x_{ij},\;\;\text{for}\;k=1,2,\ldots ,K-1\;\beta_{j}|\lambda ,\gamma_{j}\sim \left(1-\gamma_{j}\right)\times \mathrm{DE}\left(0,1/\lambda_{0}\right)+\gamma_{j}\times \mathrm{DE}(0,1/\lambda ),\;\;\text{for}\;j=1,\ldots ,p\;\lambda \sim \mathrm{Gamma}(a,b)\;\alpha_{k}\sim \mathrm{Normal}\left(0,\sigma_{\alpha_{k}}^{2}\right),\alpha_{1}<\alpha_{2}<\ldots <\alpha_{K-1},\;\;\text{for}\;\;k=1,2,\ldots ,K-1\;\gamma_{j}\sim \mathrm{Bernoulli}\left(\pi_{j}\right),\;\;\text{for}\;j=1,\ldots ,p\;\pi_{j}=t\;\text{or}\;\pi_{j}\sim \mathrm{Beta}(c,d),\;\;\text{for}\;j=1,\ldots ,p$$

When fitting this model the user is required to specify the parameter for the spike (*λ*_0_) using lambda0, which should be a large positive value (e.g., 20), while the slab is taken to be a double exponential distribution with parameter *λ* where that *λ* is from a Gamma distribution with parameters a and b. As with the regression-based variable inclusion indicator and Normal spike-and-slab models, the variable inclusion indicator *γ*_*j*_ is assumed to follow a Bernoulli distribution with parameter *π*_*j*_. The user can use either a fixed constant prior (default) or a random prior. When using a fixed constant prior, the user must specify both gamma.ind=“fixed” and set pi.fixed to some constant in the (0, 1) interval (default is 0.05). Alternatively, a random prior for *π*_*j*_ is achieved by specifying both gamma.ind=“random” and parameter values (c.gamma and d.gamma) for the Beta distribution. If unpenalized coefficients are included in the model, their coefficients are $\zeta \sim \mathrm{Normal}\left(0,\sigma_{coerce\;}^{2}\right)$.

### 2.6. Other Package Functions

The ordinalbayes function yields an object of class ordinalbayes. Generic functions have been specifically tailored to extract meaningful results from the resulting MCMC chain. The print function returns several summaries from the MCMC output for each parameter monitored, including: the 95th lower confidence limit for the highest posterior density (HPD) credible interval (Lower95), the median value (Median), the 95th upper confidence limit for the HPD credible interval (Upper95), the mean value (Mean), the sample standard deviation (SD), the mode of the variable (Mode), the Monte Carlo standard error (MCerr,) percent of SD due to MCMC (MC%ofSD), effective sample size (SSeff), autocorrelation at a lag of 30 (AC.30), and the potential scale reduction factor (psrf). The plot function provides a trace of the sampled output and optionally the density estimate for each variable in the chain. This function additionally adds the appropriate beta and gamma labels for each penalized variable name.

When identifying important covariates, the regression-based variable inclusion indicator, normal spike-and-slab, and double exponential spike-and-slab Bayesian ordinal models all incorporate a variable inclusion indicator, *γ*_*j*_, in the model. Variable selection can be based on whether the posterior mean of *γ*_*j*_ exceeds a pre-specified threshold. Alternatively, we can use the Bayes factor to test the hypotheses *H*_0*j*_ : *γ*_*j*_ = 0 versus *H*_*aj*_ : *γ*_*j*_ = 1, where the null hypothesis is rejected for feature *j* if the Bayes factor exceeds a pre-specified threshold. For the LASSO, normal spike-and-slab, and double exponential spike-and-slab Bayesian ordinal models, the Bayes factor can be used to test an interval null hypothesis *H*_0*j*_ : |*β*_*j*_| ≤ *ϵ* versus *H*_*aj*_ : |*β*_*j*_| > *ϵ*, where *ϵ* is a small positive value that is close to 0. For the regression-based variable inclusion indicator Bayesian ordinal model, the Bayes factor can be used to test *H*_0*j*_ : |*γ*_*j*_*β*_*j*_| ≤ *ϵ* versus *H*_*aj*_ : |*γ*_*j*_*β*_*j*_| > *ϵ*. Note that for the Bayesian LASSO, no variable inclusion indicators are incorporated, so variable selection can only be performed using the Bayes factor for *β*. The summary function requires an ordinalbayes object, and the user can specify epsilon (default 0.1) for testing the null hypothesis that *H*_0*j*_ : |*β*_*j*_| ≤ *ϵ*. The output from summary is a list containing the following components: alphamatrix, the MCMC output for the threshold parameters; betamatrix, the MCMC output for the penalized parameters; zetamatrix, The MCMC output for the unpenalized parameters (if included); gammamatrix, the MCMC output for the variable inclusion parameters (not available when model = “lasso”); gammamean, the posterior mean of the variable inclusion indicators (not available when model = “lasso”); gamma.BayesFactor, Bayes factor for the variable inclusion indicators (not available when model = “lasso”); Beta.BayesFactor, Bayes factor for the penalized parameters; and lambdamatrix, the MCMC output for the penalty parameter (not available when model=“normalss”). The coef function also accepts an ordinalbayes object and returns a function (default is method=mean) of the posterior distribution of the penalized parameter estimates and variable inclusion indicators.

The predict function accepts an ordinalbayes object and optionally allows the user to specify new data for unpenalized predictors and the penalized predictors by invoking neww = and newx =, respectively. If neww and newx are not supplied, the original data are used for prediction. The model.select parameter allows the user to obtain model predictions through one of three different methods. When model.select = “average” (default), the mean coefficient values over the MCMC chain are used to estimate fitted probabilities; the predicted response is attaining the maximum fitted probability. When model.select = “median”, the median coefficient values over the MCMC chain are used to estimate fitted probabilities; the predicted ordinal response is attaining the maximum fitted probability. When model.select = “max.predicted.class”, each step in the chain is used to calculate fitted probabilities and the ordinal response, then the final predicted ordinal response is taken as that ordinal response level that is most frequently predicted. The function fitted is synonymous with predict.

### 2.7. Analysis of Cervical Cancer Dataset

We downloaded the transcript-level HTSeq count data for the 309 subjects from the The Cancer Genome Atlas Cervical Squamous Cell Carcinoma and Endocervical Adenocarcinoma (TCGA-CESC) project [21] having transcriptome profiling performed using the TCGAbiolinks Bioconductor package [22]. We then restricted attention to the 253 cervical cancer subjects with a primary diagnosis of squamous cell carcinoma. Subsequently, we removed one subject whose sample was FFPE preserved, one subject with metastatic disease, two subjects who contributed only solid normal tissue, and seven subjects lacking FIGO stage. This left 242 subjects in Stage I (*N* = 124), II (*N* = 61), and III-IV (*N* = 57). Using the DESeq2 Bioconductor package [23], we performed differential expression analysis using the stage as the independent predictor in the negative binomial model. We then applied the regularized log transformation to robustly transform the count data to a log_2_ scale to stabilize the variance and then filtered the resulting dataset to retain transcripts that had a mean expression > 0.5 and FDR< 0.10 from the stage I versus stages III/IV contrast.

We fit a regression-based variable inclusion indicator Bayesian ordinal model using a Beta(0.01, 0.19) hyperprior for the *π*_*j*_ using the runjags package to run three parallel chains with 5000 burn-in, 5000 tuning steps, and thinned to keep every third step in the sampling process to reduce auto-correlation in our posterior samples, and we kept 9999 saved steps per chain. Convergence was assessed using Gelman and Rubin’s potential scale reduction factor (PSRF).

## 3. Results

There were 1137 transcripts that were differentially expressed at a Benjamini–Hochberg FDR< 0.05 and 2009 transcripts that were differentially expressed at a Benjamini-Hochberg FDR< 0.10 when examining the contrast between stage I and stages III/IV. These 2009 transcripts were retained for Bayesian modeling. Forty transcripts had a Bayes factor > 4 when testing *H*_0*j*_ : |*γ*_*j*_*β*_*j*_| ≤ 0.1 versus *H*_*aj*_ : |*γ*_*j*_*β*_*j*_| > 0.1. Forty-one transcripts had a Bayes factor > 4 when testing *H*_0*j*_ : *γ*_*j*_ = 0 versus *H*_*aj*_ : *γ*_*j*_ = 1 (Table 1). Notably, the features were the same with the exception that Bayes factor testing *γ*_*j*_ = 0 additionally identified ENSG00000115548 (Gene symbol *KDM3A*).

Many genes listed in Table 1 are relevant to cervical cancer, related cancers of the female reproductive system, or cancer in general. For example, in a tissue-based study, *CAPN6* was not detected in normal cervical squamous epithelium, but its expression was observed in low-grade and increased further in high-grade squamous cervical intraepithelial lesions [24]. *KDM3A* is an epigenetic regulator that has been found to be highly expressed in cervical cancer tissues and involved in cervical cancer progression [25]. *P4HA1* was included in a five-gene signature to predict cervical cancer prognosis [26]. A previous study suggested that *CMTM5* is a tumor suppressor that is frequently methylated and thus loses function in cancer [27], including cervical cancer [28]. *RAB6C* has been shown to be aberrantly methylated in cervical cancer compared to normal tissues [29]. *ALDH7A1* was among 30 genes that demonstrated a dose–response pattern with NNK, a tobacco carcinogen, in cervical cancer samples [30], implicating tobacco may be a causative factor in cervical cancer development in addition to HPV infection.

Other genes, while not yet described in cervical cancer, have been found to be prognostic in ovarian cancer (*RGS11* [31], *CHAD* and *CBLN2* [32], *NETO1* [33], *HSPE1* [34], and *BIRC6*, which Lnc-TTC27–9 is intronic to [35]). The expression of *SH3BP5* is reduced in ovarian cancer samples compared to normal tissue and that silencing of Sab protein expression may lead to chemo-resistance [36]. The expression of *SNTN* has high discriminatory power to differentiate between normal tissue, serous borderline ovarian tumors, and serous ovarian carcinoma [37]. *IL22RA2* is highly expressed in various tissues, including those in the female reproductive system [38]. With respect to genes associated with other cancers, *NXT2* was among 12 genes used to define prognostic risk groups in melanoma [39]. A review article described that the aberrant expression of *HS3ST3B1* is observed in many cancers, and the authors posited that *HS3ST3B1* may act as a tumor-promoting enzyme [40]. The expression of *KRT85* was found to be associated with overall survival in subjects with colon cancer [41].

When using the fitted model using the 2009 transcripts, only 16.9% of subjects were misclassified, with all misclassifications in Stage II. However, when fitting a parsimonious model including only the 41 transcripts in Table 1, the misclassification rate decreased to 11.6%. For evaluating the effectiveness of this multi-category classification, we evaluated the hypervolume under ROC manifold [42,43], which was 0.865 (95% CI: 0.800, 0.914) for the 41 transcript model, indicating good discrimination among the three stages.

## 4. Discussion

The ordinalbayes package is based on runjags and enables the user to easily fit penalized ordinal Bayesian cumulative logit models to high-dimensional datasets. The package includes methods for monitoring the mixing of chains (plot) and convergence (print). It also includes a summary function that permits the user to estimate the Bayes factor for testing an interval null hypothesis for *β*_*j*_ and for testing the null that *γ*_*j*_ = 0 to assist the user with variable selection. The coef function uses the posterior distribution to return summary estimates of the penalized *β*_*j*_ and the *γ*_*j*_ indicators. The predict (or equivalently, fitted) function can be used to obtain the estimated ordinal response probabilities as well as the predicted ordinal response level for each observation.

When applied to The Cancer Genome Atlas cervical cancer dataset, predictive performance was excellent. When restricting attention to only the 41 transcripts with a Bayes factor > 4, predictive performance yielded an overall misclassification error of 11.6%, though the misclassification error increased from 0% for Stage I and III/VI in the full model to 3.2% and 14.0%, respectively, in the reduced model. Interestingly, transcripts that were identified have known associations with cervical cancer, cancers of the female reproductive system, and other cancer in general. The syntax we used to analyze this dataset appears in the Appendix B.

## Figures and Tables

**Table 1.** Transcripts significant from the regression-based variable inclusion indicator Bayesian ordinal model when testing *H*_0*j*_ : *γ_j_* = 0 versus *H_aj_* : *γ_j_* = 1 using the Bayes factor and a threshold of 4. Annotation information obtained on 28 February 2022 from https://www.ncbi.nlm.nih.gov/gene, https://www.genecards.org, and https://lncipedia.org.

Ensemble ID	Gene Symbol	Chr	$\bar{\gamma }$

ENSG00000076344	*RGS11*	16	0.179
ENSG00000077274	*CAPN6*	X	0.264
ENSG00000101888	*NXT2*	X	0.194
ENSG00000115548	*KDM3A*	2	0.174
ENSG00000122884	*P4HA1*	10	0.186
ENSG00000125430	*HS3ST3B1*	17	0.286
ENSG00000131370	*SH3BP5*	3	0.175
ENSG00000135443	*KRT85*	12	0.334
ENSG00000136457	*CHAD*	17	0.179
ENSG00000138398	*PPIG*	2	0.240
ENSG00000150636	*CCDC102B*	18	0.281
ENSG00000161277	*THAP8*	19	0.283
ENSG00000163510	*CWC22*	2	0.301
ENSG00000164485	*IL22RA2*	6	0.196
ENSG00000164651	*SP8*	7	0.231
ENSG00000166091	*CMTM5*	14	0.215
ENSG00000166342	*NETO1*	18	0.197
ENSG00000171121	*KCNMB3*	3	0.186
ENSG00000177173	Pseudogene, parent *NAP1L4P1*	1	0.258
ENSG00000180229	*HERC2P3*	15	0.196
ENSG00000188817	*SNTN*	3	0.236
ENSG00000197360	*ZNF98*	19	0.214
ENSG00000203601	LINC00970	1	0.316
ENSG00000225449	*RAB6C-AS1*	2	0.235
ENSG00000230201	Pseudogene, parent *ATP6V0CP1*	17	0.286
ENSG00000233996	Pseudogene, parent *KDM3AP1*	2	0.248
ENSG00000236138	*DUX4L26*	3	0.247
ENSG00000236819	LINC01563	17	0.311
ENSG00000250602	lnc-ALDH7A1-1	5	0.246
ENSG00000253923	Pseudogene, parent *HSPE1*	8	0.302
ENSG00000256980	*KHDC1L*	6	0.207
ENSG00000259083	lnc-TRAPPC6B-1	14	0.263
ENSG00000259134	LINC00924	15	0.352
ENSG00000260484	lnc-OPRK1-2	8	0.263
ENSG00000263612	lnc-ZNF517-4	8	0.228
ENSG00000264049	MIR4737	17	0.266
ENSG00000264954	*PRR29-AS1*	17	0.221
ENSG00000265579	lnc-CBLN2-1	18	0.227
ENSG00000271711	Pseudogene, parent *SAP30*	3	0.264
ENSG00000272071	lnc-PAPD7-2	5	0.279
ENSG00000276517	Lnc-TTC27-9	2	0.221

## Data Availability

The TCGA-CESC high-throughput gene expression data can be downloaded using the TCGAbiolinks Bioconductor package.

## Appendix A. Example

The ordinalbayes R package is available from CRAN and github (https://github.com/kelliejarcher/ordinalbayes), where the latter includes installation instructions and the example code in this Appendix. For a toy example, subset data of the cervical cancer data are stored in the data.frame named cesc. This data.frame includes age_at_index, cigarettes_per_day, race, Stage, and expression of 41 transcripts. The regression-based variable inclusion model with random prior to *π* can be fit after loading the ordinalbayes R package using the syntax:

library(“ordinalbayes”)
data(“cesc”)
fit <- ordinalbayes(Stage~1, data = cesc,
x = cesc[,5:45], model = “regressvi”,
gamma.ind = “random”, c.gamma = 0.01, d.gamma = 0.19,
seed = 26, adaptSteps=2000, burnInSteps=2000, numSavedSteps = 3000)

This took 2.93 min on a 13 inch MacBook Pro with four cores and 16GB RAM. To include age_at_index and cigarettes_per_day as unpenalized covariates while including the 41 gene expression covariates as penalized covariates, the syntax is

fit.unpenalized <- ordinalbayes(Stage~age_at_index + cigarettes_per_day,
data = cesc, x = cesc[,5:45], model = “regressvi”,
gamma.ind = “random”, c.gamma = 0.01, d.gamma = 0.19,
seed = 26, adaptSteps=2000, burnInSteps=2000, numSavedSteps = 3000)

You can evaluate various aspects of the MCMC results of the ordinalbayes object by issuing the print command.

print(fit)

including the psrf to assess model convergence. Please note that to foster reproducibility of our output, we set the random seed. Subsequent runs using different seeds will produce different results due to the random nature of the MCMC sampling. To summarize the fitted model object,

summary.fit<-summary(fit)

To identify which transcripts had a Bayes factor > 4 when testing *H*_0*j*_ : |*γ*_*j*_*β*_*j*_| ≤ 0.1 versus *H*_*aj*_ : |*γ*_*j*_*β*_*j*_| > 0.1,

names(which(summary.fit$Beta.BayesFactor>4))

Similarly, to identify which transcripts had a Bayes factor > 4 when testing *H*_0*j*_ : *γ*_*j*_ = 0 versus *H*_*aj*_ : *γ*_*j*_ = 1,

names(which(summary.fit$gamma.BayesFactor>4))

To obtain the $\bar{\gamma }$ estimates we used the following code:

coefficients<-coef(fit)
coefficients$gamma[which(summary.fit$gamma.BayesFactor>4)]

To obtain model predictions,

phat<-predict(fit)
table(phat$class, cesc$Stage)

**Table:**

	1	2	3
1	120	15	2
2	4	32	13
3	0	14	42

## Appendix B. Reproducing CESC Results Using Bioconductor Objects

The data used in this example are stored in the finalSet object. Because this object was derived using the DESeq2 BioConductor package, we load it first. Please note that due to the use of the default parameters for the number of saved steps per chain (9999) and the large size of this dataset, the model took 3.2 days to run on a 13 inch MacBook Pro with four cores and 16GB RAM. For those interested in running examples using this package, a smaller version of these data, reducedSet, which includes the 41 transcripts, may be used instead. Alternatively, parameters related to the number of steps can be reduced.

The regression-based variable inclusion model with random prior to *π* was fit after loading the ordinalbayes R using the syntax:

library(“DESeq2”)
library(“ordinalbayes”)
data(finalSet)
fitted.regressvi.random<-ordinalbayes(Stage~1, data=colData(finalSet),
x=t(assay(finalSet)), model=“regressvi”,
gamma.ind=“random”, c.gamma=0.01, d.gamma=0.19, seed=26)

Again note that to foster reproducibility of our output, we set the random seed. Subsequent runs using different seeds will produce different results due to the random nature of the MCMC sampling.

We then summarize the fitted model object and to identify which transcripts had a Bayes factor > 4 when testing *H*_0*j*_ : |*γ*_*j*_*β*_*j*_| ≤ 0.1 versus *H*_*aj*_ : |*γ*_*j*_*β*_*j*_| > 0.1,

summary.model.fit<-summary(fitted.regressvi.random)
names(which(summary.model.fit$Beta.BayesFactor>4))

Similarly, to identify which transcripts had a Bayes factor > 4 when testing *H*_0*j*_ : *γ*_*j*_ = 0 versus *H*_*aj*_ : *γ*_*j*_ = 1,

names(which(summary.model.fit$gamma.BayesFactor>4))

To obtain the $\bar{\gamma }$ estimates, we used the following code:

coefficients<-coef(fitted.regressvi.random)
coefficients$gamma[which(summary.model.fit$gamma.BayesFactor>4)]

To obtain model predictions,

phat<-predict(fitted.regressvi.random)
table(phat$class, colData(finalSet)$Stage)

**Table:**

	1	2	3
1	124	28	0
2	0	20	0
3	0	13	57

To determine the adequacy of a more parsimonious model, we then restricted attention to 41 transcripts having gamma.BayesFactor>4. The reducedSet object is provided in the ordinalbayes package; however, due to the random nature of the MCMC sampling, the number of transcripts with a Bayes factor for *γ* could differ, so we demonstrate how we derived our object.

reducedSet<-finalSet[which(summary.model.fit$gamma.BayesFactor>4),]
fitted.regressvi.reduced<-ordinalbayes(Stage~1, data=colData(reducedSet),
x=t(assay(reducedSet)), model=“regressvi”,
gamma.ind=“random”, c.gamma=100, d.gamma=1, seed=26)

Because we were using gamma.ind=“random”, we changed the parameter values for the variable inclusion indicator hyperprior to c.gamma=100, d.gamma=1 ensure virtually all transcripts would be included in each model. If fitting a model using gamma.ind=“fixed”, the hyperprior pi.fixed=0.99 would accomplish the same thing. This smaller model only took 9.1 min to complete.

phat.reduced<-predict(fitted.regressvi.reduced)
table(phat.reduced$class, colData(reducedSet)$Stage)

**Table:**

	1	2	3
1	120	9	1
2	4	45	7
3	0	7	49

This more parsimonious model that included 41 transcripts had a misclassification rate of 11.6%. The class-specific misclassification rates [Stage I (3.2%), Stage II (26.2%), Stage III/IV (14.0%)] may indicate that smaller classes are more difficult to predict. To obtain the hypervolume under the ROC manifold, we load the mcca R package [43] and use the hum function and estimate the 95% confidence interval using the bootstrap method.

library(“mcca”)
hum.fit <- hum(colData(reducedSet)$Stage, phat.reduced$predicted,
method = “prob”)
hum.fit
hum.ci <- ests(y = colData(reducedSet)$Stage, d = phat.reduced$predicted,
acc = “hum”, level = 0.95, method = “prob”)
hum.ci
$value
[1] 0.8645291
$se
[1] 0.02955225
$interval
[1] 0.7996765 0.9144449

The mcca R package additionally includes a plot function that can be used to explore the plot of the three-dimensional ROC surface.

## Appendix C. User-Defined Parameters in Ordinalbayes Function

**Table A1.** ordinalbayes parameters available for all models.

Parameter	Description and Default Values

alpha.var	Variance for *α_k_* in the MCMC chain (default 10)
coerce.var	Variance associated with any unpenalized predictors in the MCMC chain (default 10)
adaptSteps	Number of iterations for adaptation (default 5000)
burnInSteps	Number of iterations of the Markov chain to run (default 5000)
nChains	Number of parallel chains to run (default 3)
numSavedSteps	Number of saved steps for each chain (default 9999)
thinSteps	The thinning interval for monitors (default 3)
parallel	Run the MCMC on multiple processors (default TRUE)
model	Specify which penalized ordinal model to fit (default regressvi)
center	If TRUE (default), center the variables to be penalized in the model
scale	If TRUE (default), scale the variables to be penalized in the model
seed	An integer value for the random seed to ensure reproducibility
quiet	If TRUE, suppress output of JAGS (or rjags) when updating models (default FALSE)

**Table A2.** ordinalbayes parameters for each penalized ordinal Bayesian model.

Model	Parameters in Ordinalbayes Call to Specify	Description

lasso	a, b	The penalty parameter *λ* ∼Gamma(a, b) (default a = 0.1, b = 0.1)

normalss	sigma2.0	The variance for the spike (set to some small positive value, e.g., 0.01)
	sigma2.1	The variance for the slab (set to some large positive value, e.g., 10)
	gamma.ind=“fixed”, pi.fixed	Use a constant prior for *π_j_* of pi.fixed (default 0.05)
	gamma.ind=“random”, c.gamma, d.gamma	Use a random prior for *π_j_* ∼Beta(c.gamma, d.gamma), for example, c.gamma = 0.01, d.gamma = 0.19.

dess	a, b	The penalty parameter *λ* ∼Gamma(a, b) (default a = 0.1, b = 0.1)
	lambda0	The parameter value for the spike, e.g., lambda0 = 20
	gamma.ind=“fixed”, pi.fixed	Use a constant prior for *π_j_* of pi.fixed (default 0.05)
	gamma.ind=“random”, c.gamma, d.gamma	Use a random prior for *π_j_* ∼Beta(c.gamma, d.gamma), for example, c.gamma = 0.01, d.gamma = 0.19.

regressvi	a, b	The penalty parameter *λ* ∼Gamma(a, b) (default a = 0.1, b = 0.1)
	gamma.ind=“fixed”, pi.fixed	Use a constant prior for *π_j_* of pi.fixed (default 0.05)
	gamma.ind=“random”, c.gamma, d.gamma	Use a random prior for *π_j_* ∼Beta(c.gamma, d.gamma), for example, c.gamma = 0.01, d.gamma = 0.19.

## Abbreviations

The following abbreviations are used in this manuscript:

MDPI: Multidisciplinary Digital Publishing Institute
FIGO: International Federation of Gynecology and Obstetrics
TCGA: The Cancer Genome Atlas Project
CRAN: Comprehensive R Archive Network
HPV: Human Papilloma Virus
LASSO: Least Absolute Shrinkage and Selection Operator
MCMC: Markov Chain Monte Carlo
DE: Double exponential
PSRF: potential scale reduction factor
HTSeq: High-throughput sequencing
FFPE: Formalin-Fixed Paraffin-Embedded
FDR: False discovery rate
